# Effect of SiO_2_ Nanoparticles on the Performance of PVdF-HFP/Ionic Liquid Separator for Lithium-Ion Batteries

**DOI:** 10.3390/nano8110926

**Published:** 2018-11-08

**Authors:** Stefano Caimi, Antoine Klaue, Hua Wu, Massimo Morbidelli

**Affiliations:** Department of Chemistry and Applied Biosciences, Institute for Chemical and Bioengineering, ETH Zurich, 8093 Zurich, Switzerland; stefano.caimi@chem.ethz.ch (S.C.); antoine.klaue@chem.ethz.ch (A.K.)

**Keywords:** lithium-ion battery, ionic-liquid-based separator, hot-pressing, inorganic nanoparticle, nanocomposite, fractal cluster

## Abstract

Safety concerns related to the use of potentially explosive, liquid organic electrolytes in commercial high-power lithium-ion batteries are constantly rising. One promising alternative is to use thermally stable ionic liquids (ILs) as conductive media, which are however, limited by low ionic conductivity at room temperature. This can be improved by adding fillers, such as silica or alumina nanoparticles (NPs), in the polymer matrix that hosts the IL. To maximize the effect of such NPs, they have to be uniformly dispersed in the matrix while keeping their size as small as possible. In this work, starting from a water dispersion of silica NPs, we present a novel method to incorporate silica NPs at the nanoscale level (<200 nm) into PVdF-HFP polymer clusters, which are then blended with the IL solution and hot-pressed to form separators suitable for battery applications. The effect of different amounts of silica in the polymer matrix on the ionic conductivity and cyclability of the separator is investigated. A membrane containing 10 wt.% of silica (with respect to the polymer) was shown to maximize the performance of the separator, with a room temperature ionic conductivity of of 1.22 mS cm${}^{-1}$. The assembled half-coin cell with LiFePO${}_{4}$ and Li as the cathode and the anode exhibited a capacity retention of more than 80% at a current density of 2C and 60 ${}^{\circ }$C.

## 1. Introduction

Recent large-scale power applications of lithium-ion batteries (LIB) including hybrid vehicles and smart grids require long cycle life, low impact on the environment and high reliability and safety [1,2,3,4,5,6]. Current technologies are based on the use of organic liquid electrolytes, which guarantee high ionic conductivity at low temperatures and long cycle stability. However, they are also highly volatile, toxic and thermally unstable and may leak out of the battery under abnormal operations [7,8,9,10,11,12,13,14]. One of the most promising alternatives to replace liquid electrolytes is the employment of ionic liquids (ILs), which possess high ion density and are characterized by high thermal stability [6,15,16,17,18,19,20,21,22,23,24]. Among the several existing ILs, those based on pyrrolidinium, and in particular Pyr13TFSI, are often considered since they exhibit low viscosities and are chemically inert towards the cell components [10,19,25,26]. The main drawback of the use of ILs is the limited ionic conductivity at low temperature, especially when mixed with the host polymer to form the separator. One possibility to increase the ionic conductivity at low temperatures is the addition of inorganic nanoparticles (NPs) such as SiO${}_{2}$, Al${}_{2}$O${}_{3}$, TiO${}_{2}$ and CeO${}_{2}$, to the polymer matrix [27,28,29,30,31]. These fillers can improve the conductivity by reducing the polymer crystallinity and by interacting with the ionic species in the electrolyte through Lewis acid–base interactions [29,32,33,34,35]. Moreover, it is well established that the addition of inorganic NPs into the polymer matrix improves its mechanical stability, thus preventing thermal shrinkage and mechanical breakdown of the separator [35,36,37]. In order to maximize the effect of the added NPs, it is essential to disperse them in the polymer matrix uniformly and at the nanoscale level [38,39]. To achieve this, in this work, we start from a dispersion of silica NPs in water and mix it with an aqueous dispersion of PVdF-HFP NPs. The binary dispersion is then subjected to shear-driven gelation by passing through a microchannel where, if present alone, the polymer NPs undergo gelation whereas the silica NPs are shear-inactive (i.e., they are stable and do not aggregate). As the process occurs in few milliseconds the silica NPs cannot escape from the polymer gel network and remain entrapped and dispersed uniformly in the polymer matrix [40,41,42]. Poly(vinylidenefluoride-*co*-hexafluoropropylene) (PVdF-HFP) is chosen as it possesses high dielectric constant, it is chemically compatible with the electrode materials, and it is characterized by low crystallinity [27,28,39,43]. The method to form a freestanding, uniform and transparent membrane through hot-pressing, starting from the polymer/filler clusters and the IL has been developed earlier in our group and described elsewhere [44]. The effect of the presence of silica NPs in the IL-based membrane is investigated in terms of ionic conductivity and electrochemical cyclability.

## 2. Materials and Methods

### 2.1. Materials

The following chemicals have been employed without further treatments: Sodium dodecyl sulphate (SDS, purity 99%) and *N*-Propyl-*N*-Methylpyrrolidinium bis(trifluoromethane-sulfonyl)-imide (Pyr1308b, purity 99.5%) were purchased from Apollo Scientific (Bredbury, UK) and Solvionic (Toulouse, France), respectively. The water dispersion of PVdF-HFP NPs, the amorphous silica powder Tixosil 365 and bis(trifluoro methane)sulfonamide lithium salt (LiTFSI) were provided by Solvay (Bollate, Italy). The ion-exchange resin (Dowex MR-3) was purchased from Sigma-Aldrich (Steinheim, Germany). The cathode material lithium iron phosphate (LFP) is commercial grade Life Power P2 from Clariant (Muttenz, Switzerland). Before assembling, LFP and the IL-based separator were dried overnight under vacuum at 130 ${}^{\circ }$C and 60 ${}^{\circ }$C, respectively.

### 2.2. Methods

To form a suitable dispersion, Millipore water is added to the silica powder to reach a solid fraction of 30% and the mixture is mechanically stirred and repeatedly sonicated using a digital sonifier from Branson. Eventually, the dispersion is centrifuged at 2000 rpm for 10 min to remove the remaining large clusters. The binary dispersion of PVdF-HFP and SiO${}_{2}$ NPs is prepared by adding the silica NP dispersion dropwise to the PVdF-HFP NP dispersion under agitation. As the addition of SiO${}_{2}$ NPs may destabilize the PVdF-HFP dispersion due to a repartition of the adsorbed surfactant between the two species, some additional SDS is added (0.2% with respect to the solid content of the dispersion). The content of the added silica is evaluated as mass percentage with respect to the mass of polymer.

A high-shear device, HC-5000 (Microfluidics, Westwood, MA, USA), connected to a L30Z microchannel with a width of 300 μm, a rectangular cross section of 5.26·10${}^{-8}$ m${}^{2}$ and a length of 5.8 mm was used to perform shear-driven gelation of the PVdF-HFP/SiO${}_{2}$ NPs. The operating conditions for the gelation and subsequent drying to obtain the polymer-silica clusters (PSiCs) are reported elsewhere [44].

The IL solution consisted of a 0.5 M solution of LiTFSI salt dissolved into Pyr13TFSI.

The obtained clusters were mixed with the IL solution at a mass fraction of PSiC/IL equal to 30/70 wt.%, following the procedure previously reported [44]. The slurry was then transferred between two aluminum sheets in a preheated hydraulic hand-press (Rondol, Strasbourg, France) and hot-pressed at 120 ${}^{\circ }$C and 10 kN to form the separator. The cooling phase was performed while holding the pressure. The formed separator is here referred to as the PSiCIL membrane.

Small-angle light scattering (SALS) measurements were taken using a Mastersizer 2000 (Malvern, UK) equipped with a laser having a wavelength λ = 633 nm to characterize the obtained clusters in terms of their average radius of gyration, 〈*R${}_{g}$*〉, and their fractal dimension, *d${}_{f}$*, following the procedure reported elsewhere [45,46]. Measures of dynamic light scattering and zeta potential were conducted using Zetasizer Nano ZS 3600 from Malvern Instruments (Malvern, UK).

Differential scanning calorimetry (DSC) measurements were conducted using Q1000 instrument (TA Instruments, New Castle, DE, USA) using 40 μL crucibles in aluminum and a heating and cooling rate of 5 ${}^{\circ }$C min${}^{-1}$ in a nitrogen atmosphere in the temperature range from 80 to 200 ${}^{\circ }$C. The solid content is measured using a HG53 Halogen Moisture Analyzer (Mettler-Toledo, Columbus, OH, USA). Powder XRD measurements were carried out with a X’Pert PRO-MPD diffractometer (Malvern PANalytical, Malvern, UK). Data were recorded in the 5–70${}^{\circ }$ 2θ range with an angular step size of 0.05${}^{\circ }$ and a counting time of 0.26 s per step. The peaks at 2θ = 18.2, 20.0, 26.6 and 38.8 correspond to the (100), (020), (110) and (021) crystalline peaks of PVdF-HFP, respectively [47].

AC (alternating current) impedance spectroscopy was used to measure the ionic conductivity of the PSiCIL membranes using a conductivity cell consisting of two stainless steel blocking electrodes. The measurement was carried out under PEIS conditions (impedance under potentiostatic mode), Δ*V* = 5 mV and frequency range from 300 kHz to 1 Hz. The resistance of the polymer electrolyte was measured and the ionic conductivity (σ) was obtained as follows:

(1) $$\sigma =\frac{d}{R_{b}S}$$

where *d* is the thickness of the separator, *R${}_{b}$* the bulk resistance and *S* the area of the stainless steel electrode.

SEM images were taken with a Zeiss Leo 1530 (Zeiss, Oberkochen, Germany) microscopy with a field emission gun of 5 kV and platinum coating. TEM images were performed using a Morgagni 268 from FEI equipped with a tungsten emitter operated at 100 kV.

The battery consisted of CR2032 coin cells assembled using lithium iron phosphate (LFP) and lithium metal as the cathode and the counter electrode, respectively, and the PSiCIL membrane as the separator. The electrochemical tests were performed with a Land CT2001A battery tester and with a mass loading of the active material per cell equal to 4 mg. The battery assembly was performed under argon atmosphere. The cells were cycled in the voltage range 2.5–4 V (vs. Li/Li${}^{+}$) at 60 ${}^{\circ }$C, at current densities from 0.1 to 2C. For charge/discharge performance characterization, 1C is defined as 170 mAh g${}^{-1}$.

## 3. Results and Discussion

### 3.1. Preparation of the PSiCIL Separators

The procedure to obtain the silica dispersion is reported in the experimental section. Typical properties of the obtained dispersion of SiO${}_{2}$ NPs in water are summarized in Table 1.

**Figure 1:**
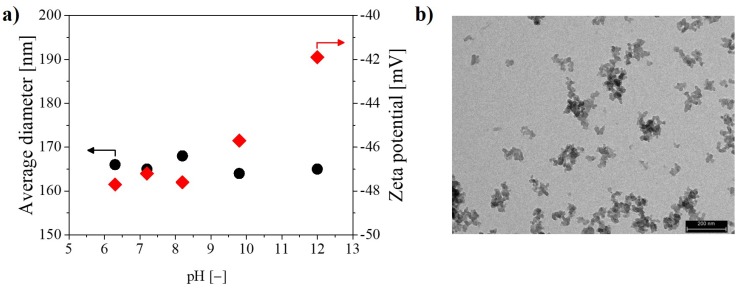
(**a**) Average diameter and zeta potential of the silica NPs as a function of pH in the silica dispersion. (**b**) TEM image of the dried silica NPs (scale 200 nm).

For the application at hand, the silica NPs have to be well-dispersed in water and should be negatively charged to maintain their stability while mixed with the negatively charged PVdF-HFP NPs. As reported in Table 1, these requirements are met by the silica NPs, showing an average diameter smaller than 200 nm and a negative potential larger than 40 mV (absolute value). In order to investigate the effect of pH, Figure 1a reports the average diameter and the zeta potential of the silica NPs in the pH range 6–12. To better appreciate the morphology of the silica NPs, Figure 1b reports a TEM image of the dried silica dispersion. From Figure 1a, it is seen that the dispersed SiO${}_{2}$ NPs have a constant average diameter smaller than 200 nm and are negatively charged with the zeta potential ranging from −48 to −42 mV. Moreover, from Figure 1b, it is possible to recognize that the each silica NP is a nanocluster made of 10 nm silica primary particles.

The chemical and physical properties of the used PVdF-HFP NP dispersion are reported in the Supplementary Materials (Table S1). The anionic surfactant was extracted from the polymer dispersion by repeated washing with ion-exchange resin Dowex MR-3 to reduce the colloidal stability of the polymer NPs and facilitate the gelation under shear.

**Figure 2:**
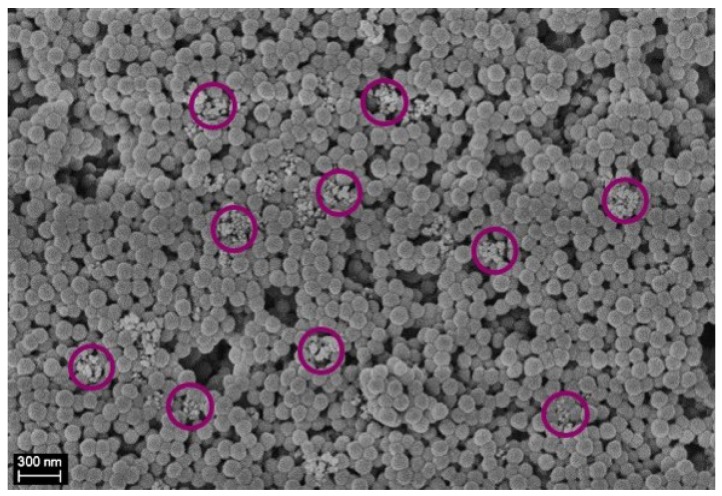
SEM picture of the silica NPs entrapped in the polymer matrix after a single passage through the microchannel (scale 300 nm). Some silica NPs are encircled in violet to be better visualized.

The polymer and filler dispersions are mixed as described in the experimental section and the binary system is subjected to intense shear by forcing it to pass through a microchannel so as to have rapid gelation of the polymer NPs with the typical fractal characteristics. Since the formation of the polymer gel network occurs in few milliseconds and the silica NPs are shear-inactive (i.e., they do not aggregate under the given shear rate), the fillers, silica NPs, have no time to escape and remains entrapped in the formed matrix at a nanoscale level. In order to have complete capture of the silica nanoparticles, it is of utmost importance to obtain a compact gel after a single passage through the microchannel, as discussed elsewhere [40,48]. This depends on the solid content of the polymer/silica dispersion: the higher the solid content, the greater the compactness of the formed gel. In order to analyze the distribution of the silica NPs inside the polymer matrix, SEM pictures of the gel obtained from the microchannel are shown in Figure 2.

**Figure 3:**
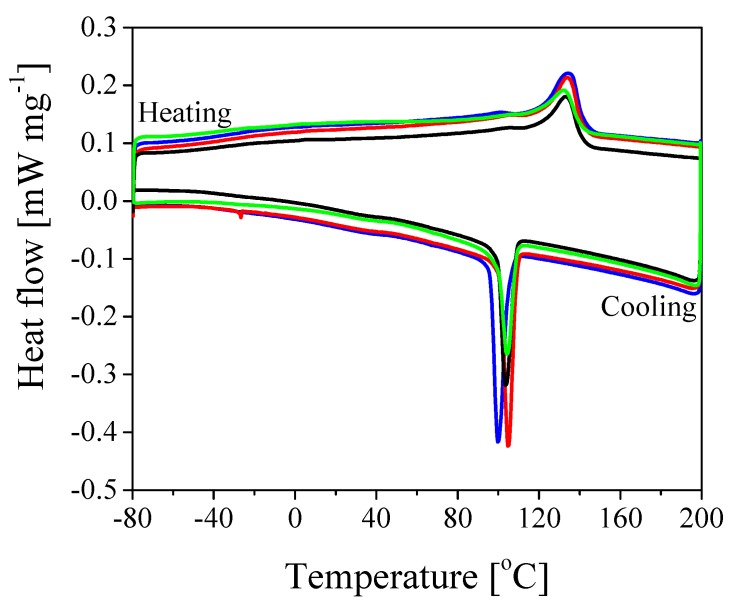
DSC of the composite gel obtained after one passage through the microchannel at different percentages of the fillers, silica NPs. Blue curve: PVdF-HFP; Red curve: PVdF-HFP + 10% SiO${}_{2}$; Black curve: PVdF-HFP + 20% SiO${}_{2}$; Green curve: PVdF-HFP + 30% SiO${}_{2}$.

From Figure 2 it is evident that the shear-induced gelation is capable of dispersing uniformly and randomly the silica NPs as fillers within the polymer matrix, where the silica NPs with respect to the PVdF-HFP NPs are clearly and easily distinguishable (whiter and smaller ones, encircled in violet in Figure 2). It is worth noticing that the fillers are uniformly dispersed at the nanoscale level (i.e., the silica NPs are smaller than 200 nm). Moreover, it is possible to observe that also the PVdF-HFP NPs maintain their original identity. In order to investigate the physical properties of the pure polymer and of the composite material, DSC is performed. Figure 3 reports the results of the heating and cooling ramps of the dried gel at different contents of SiO${}_{2}$. The melting and crystallization temperatures as well as the crystallinity of the composite derived from the DSC heating and cooling curves are reported in Table 2.

The results reported in Table 2 show that the amount of SiO${}_{2}$ NPs affects only slightly the melting and crystallization temperatures. The melting temperature of the pure polymer PVdF-HFP results in approximately 134 ${}^{\circ }$C as derived from the maximum of the heating curve and progressively decreases with increasing amount of SiO${}_{2}$ to reach approximately 131 ${}^{\circ }$C at 30 wt.% content of silica. The crystallization temperature, on the other hand, is measured from the minimum of the cooling curve, which is approximately 98 ${}^{\circ }$C for the pure polymer and 100 ${}^{\circ }$C for the composite material, independently of the amount of silica. Furthermore, the enthalpy of melting and the crystallinity significantly decrease with increasing content of SiO${}_{2}$ NPs. These results are expected and are related to the hindered reorganization of the polymer chains due to the cross-linking centers formed by the interaction of Lewis acid groups with the polar groups (i.e., the -F atoms of the polymer chains). This interaction can stabilize the amorphous structure and facilitates the transport of Li${}^{+}$ ions (i.e., the ionic conductivity), as observed by several authors [29,33,34,49,50].

**Figure 4:**
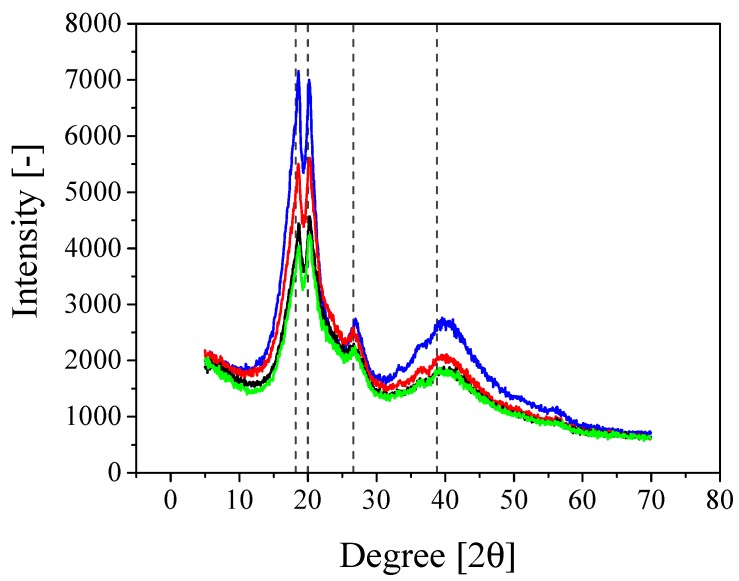
XRD of the composite gel obtained after one passage through the microchannel at different percentages of the fillers, silica NPs. Blue curve: PVdF-HFP; Red curve: PVdF-HFP + 10% SiO${}_{2}$; Black curve: PVdF-HFP + 20% SiO${}_{2}$; Green curve: PVdF-HFP + 30% SiO${}_{2}$.

In order to measure the crystallinity of the polymer and to investigate the effect of the introduction of the SiO${}_{2}$ NPs on it, XRD measurements are performed and the results are shown in Figure 4.

It is seen that the spectrum of the pure polymer confirms the partial crystallization of PVdF units in the copolymer and gives a semi-crystalline structure of PVdF-HFP [27]. Moreover, the crystallinity of the polymer has been considerably reduced upon the addition of silica NPs. The intensity of the crystalline peaks, indeed, decreases and broadens when increasing the amount of SiO${}_{2}$. This reduction in crystallinity is attributed to the changes of the chain conformation due to the presence of the silica NPs, which again facilitate higher ionic conduction [51,52].

As described in the experimental section, the dried PSiCs containing different percentages of SiO${}_{2}$ are mixed with the IL solution at a mass fraction PSiC/IL equal to 30/70 wt.% and left at rest overnight to allow full impregnation of the pores of the PSiCs by the IL solution, before being hot-pressed. After the hot-pressing, it is possible to obtain a freestanding, homogeneous and transparent 50-μm thick IL-based membrane (referred as PSiCIL membrane) containing the silica NPs at a nanoscale level. A SEM picture showing the preservation of the internal morphology after hot-pressing has been reported in our previous work [44]. To prevent water absorption, the PSiCIL membranes are stored in a nitrogen atmosphere.

### 3.2. Electrochemical Properties of the PSiCIL Separators

**Figure 5:**
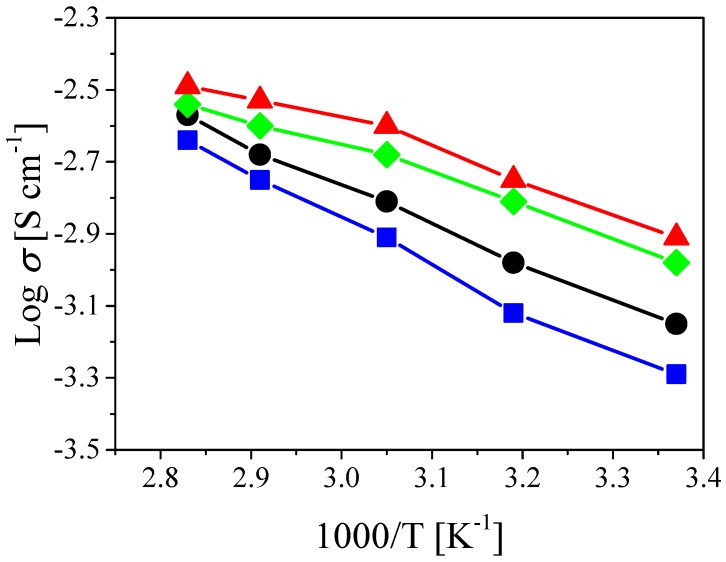
Ionic conductivity at 25, 40, 55, 70 and 80 ${}^{\circ }$C of the PSiCIL membranes containing 70 wt.% of IL and 0% (blue squares) [44], 5% (green diamonds), 10% (red triangles), and 15% (black circles) of SiO${}_{2}$, respectively.

The ionic conductivity of the PSiCIL membranes was measured by AC impedance spectroscopy in the temperature range from 25 to 80 ${}^{\circ }$C, at increasing percentages of silica (with respect to the polymer) in the PSiC, and the results are reported in Figure 5 and Table S2 in the Supplementary Materials. As can be seen in Figure 5, the ionic conductivity improves as the temperature increases. This can be understood by taking into account the effect of the temperature on the viscosity of the IL, which decreases as the temperature rises. The introduction of SiO${}_{2}$ NPs within the polymer matrix substantially improves the ionic conductivity of the PSiCIL membrane. In particular, the room temperature conductivity increases from 0.51 mS cm${}^{-1}$ for the pure polymer to 1.04, 1.22 and 0.71 mS cm${}^{-1}$ with 5%, 10%, and 15% of SiO${}_{2}$, respectively. The same trend is observed in the entire temperature range, where the membrane containing 10% of SiO${}_{2}$ NPs shows the highest values of ionic conductivity reaching 1.77, 2.51, 2.95 and 3.23 mS cm${}^{-1}$ at 40, 55, 70 and 80 ${}^{\circ }$C, respectively (the conductivity data for the other samples are summarized in Table S2 in the Supplementary Materials). Interestingly, it is observed that the increase in conductivity is not a linear function of the SiO${}_{2}$ content. The conductivity values corresponding to the SiO${}_{2}$ content of 15% are lower than those at an SiO${}_{2}$ content of 5% and 10%. This behavior has been previously observed in the literature and attributed to the fact that at low filler concentrations the interaction between polymer matrix and SiO${}_{2}$ NPs facilitates the transport of Li${}^{+}$ ions. However, when the SiO${}_{2}$ concentration reaches a certain level, the dilution effect predominates and the ionic conductivity decreases [27,53]. As reported by Stephan et al. [27], the highest conductivity is reached when the filler content ranges from 8 to 10 wt.%. The values of the ionic conductivity obtained in this work are in line with those previously reported in the literature for polymer/IL separators [44,54].

**Figure 6:**
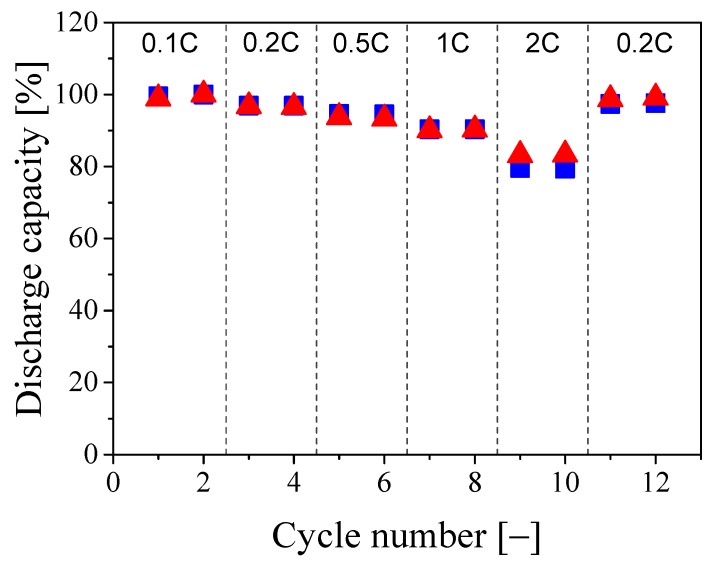
Discharge capacity, normalized with respect to the initial cycle at 0.1C, at 60 ${}^{\circ }$C of the PSiCIL membranes containing 70 wt.% of IL with 0% (blue squares) and 10% (red triangles) of SiO${}_{2}$, respectively.

The membrane with an SiO${}_{2}$ content of 10%, showing the highest ionic conductivity, was used as separator to assemble CR2032 coin cells, with LFP and lithium metal as electrodes. The corresponding discharge capacity was measured as a function of the applied current density and compared in Figure 6 with the values obtained using the membrane made of the PVdF-HFP/IL composite without silica [44]. It is seen that the discharge capacities of the two membranes are almost identical up to a current density of 1C, with a capacity retention higher than 90% with respect to the initial cycles at 0.1C. At a current density of 2C, a clear difference between the two membranes is observed, where the normalized discharge capacity is 79.5% and 83.3% for the separators with 0 and 10% of SiO${}_{2}$, respectively. Such a superior high capacity retention can be attributed to the fractal structure of the polymer clusters and to the bicontinuous morphology of the separator. Indeed, the high and well-controlled porosity formed via shear-gelation is preserved during the hot-pressing phase, where the IL solution fully impregnates the pores forming a multitude of channels through which the ions can flow, thus showing limited loss of capacity at high current density. On the other hand, the positive contribution of the silica NPs can be attributed to two factors. Firstly, the silica NPs might hinder the reorganization, even if already limited, of the polymer particles during the membrane formation process, thus leaving more channels open to the ions transfer. Secondly, as discussed in the previous paragraph, the performance at high C-rates are improved because of the same silica/polymer interactions which favor the ionic conductivity. At low current densities this is not observed as the existing channels, given the fractal geometry of the polymer clusters, are well-developed and the ion transfer is not limited by them. It is worth mentioning that after the cycles at 2C, the battery is tested again at 0.2C showing a recovery for the membranes containing 0 and 10% of SiO${}_{2}$ of 97.5% and 99%, respectively, thus showing limited performance loss during the cycles at high current densities. It is also worth pointing out that the cycle efficiency remained close to unity during all cycles. Moreover, the very low dependence of the discharge capacity on the applied current density is not commonly observed when considering earlier literature results [36,52,55].

## 4. Conclusions

In this work, we have analyzed the effect of dispersing silica NPs into PVdF-HFP/IL membranes on the ionic conductivity and discharge capacity of lithium-ion batteries. In particular, starting from the corresponding powder, we have formed a stable water dispersion of silica NPs, which could be mixed with a PVdF-HFP NP dispersion, to form a binary dispersion which was then subjected to intense shear-driven gelation. As the gelation occurs extremely fast, the silica NPs cannot escape during the gel network formation and remain entrapped and dispersed into the polymer matrix at the nanoscale level. The introduction of silica NPs into the polymer matrix was shown via DSC and XRD to reduce the crystallinity of the polymer, thus stabilizing the amorphous structure and facilitating the transport of Li${}^{+}$ ions.

The so-produced PVdF-HFP-SiO${}_{2}$ composite clusters (PSiCs) were mixed with an IL solution and hot-pressed to form a membrane, so as to analyze the effect of the silica NPs on its electrochemical performance. It was observed that the ionic conductivity increases as the SiO${}_{2}$ content increases. The ionic conductivity reaches a maximum at an SiO${}_{2}$ content of 10%, being 1.22 mS cm${}^{-1}$ at room temperature, and then decreases as the SiO${}_{2}$ content further increases. The membrane formed with 10% SiO${}_{2}$ was used to assemble coin cells and tested for cyclability at different C-rates at 60 ${}^{\circ }$C. At low current densities, no significant differences between the membranes with 0% and 10% silica were observed and the measured discharge capacities at 1C were higher than 90% of the ones measured at 0.1C, showing excellent capacity retention even at high current densities. At 2C, the membrane containing 10% silica performed better, showing a discharge capacity of 83.3%, compared to 79.5% of the membrane containing no silica. This can be attributed to the positive effect of the dispersed SiO${}_{2}$ NPs, which, on one side hinder the reorganization of the polymer NPs, thus reducing the crystallinity and increasing the amorphous phase, and on the other side, favor the transfer of ions because of their interaction with the polymer matrix.

## Figures and Tables

**Table 1:** Properties of the dispersion of SiO${}_{2}$ NPs in water.

	Average Clusters Diameter [nm]	PDI [−]	Zeta Potential [mV]
Tixosil 365	160	0.19	−47.9

**Table 2:** Properties of the composite material at increasing amount of SiO${}_{2}$, derived from the DSC curves in Figure 3. *T${}_{m}$*: melting temperature. *T${}_{c}$*: crystallization temperature. Δ*H${}_{m}$*: enthalpy of melting. *X${}_{c}$*: crystallinity.

	*T*${}_{m}$ [${}^{\circ }$C]	*T*${}_{c}$ [${}^{\circ }$C]	Δ*H${}_{m}$* [J g${}^{-1}$]	*X*${}_{c}$ [%]
Pure	133.6	97.6	22.19	21.2
10% SiO${}_{2}$	132.8	100	18.21	17.4
20% SiO${}_{2}$	130.5	99.7	16.92	16.2
30% SiO${}_{2}$	130.6	99.6	15.66	14.9

## Supplementary Materials

The following are available online at http://www.mdpi.com/2079-4991/8/11/926/s1, Table S1: Properties of the aqueous dispersion of PVdF-HFP NPs, Table S2: Ionic conductivity results.
