# Supplementary material for: Effect of SiO2 Nanoparticles on the Performance of PVdF-HFP/Ionic Liquid Separator for Lithium-Ion Batteries
Source: Nanomaterials (Basel). 2018 Nov 8;8(11):926. doi: 10.3390/nano8110926 (PMC6267017; doi:10.3390/nano8110926)
Supplement: Supplementary file 1 [file nanomaterials-08-00926-s001.zip › nanomaterials-378128-SI.pdf]

Supplementary Materials: Effect of SiO_2 Nanoparticles on the Performance of PVdF-HFP/Ionic Liquid Separator for Lithium-Ion Batteries

Stefano Caimi, Antoine Klaue, Hua Wu* and Massimo Morbidelli*

1. Properties of the aqueous dispersion of PVdF-HFP NPs

The PVdF-HFP nanoparticles (NPs) contain about 5 mol% of HFP and were obtained through emulsion polymerization using an anionic surfactant as the stabilizer. The properties of the latex are reported in Table S1.

Table S1. Physical and chemical properties of the aqueous dispersion of PVdF-HFP NPs.

Radius [nm]	PDI [-]	Conductivity [μS cm^{-1}]	Surface tension [mN m^{-1}]	ζ potential [mV]	pH [-]	NPs mass fraction [%]
140	0.03	920	62.70	-30	2.8	24.1

The average radius of the polymer NPs and the polydispersity index (PDI) are measured by dynamic light scattering using Zetasizer Nano ZS 3600 (Malvern Instruments). The conductivity is measured with Conductometer 712 (Metrohm) at a concentration of 5 wt.% and 25 °C. The surface tension measurements are performed with a Wilhelmy plate using DCAT 21 (Dataphysics) at a concentration of 0.5 wt.% and 25 °C. The solid content is assessed by spreading the sample over quartz sand analyzing the weight loss at 120 °C using a HG53 Halogen Moisture Analyzer (Mettler-Toledo).

2. Ionic conductivity results

Table S2. Ionic conductivity of the PSiCIL membranes in the temperature range from 25 to 80 °C at different percentages of silica (with respect to the polymer).

	Ionic conductivity [mS cm^{-1}]				
wt.% of SiO_2	25 °C	40 °C	55 °C	70 °C	80 °C
0	0.51	0.78	1.22	1.77	2.29
5	1.04	1.54	2.08	2.51	2.88
10	1.22	1.77	2.51	2.95	3.23
15	0.71	1.04	1.54	2.08	2.69
